# Why Awe Promotes Prosocial Behaviors? The Mediating Effects of Future Time Perspective and Self-Transcendence Meaning of Life

**DOI:** 10.3389/fpsyg.2019.01140

**Published:** 2019-05-29

**Authors:** Jing-Jing Li, Kai Dou, Yu-Jie Wang, Yan-Gang Nie

**Affiliations:** ^1^Research Center of Adolescent Psychology and Behavior, School of Education, Guangzhou University, Guangzhou, China; ^2^School of Marxism, Guangdong Industry Polytechnic, Guangzhou, China

**Keywords:** awe, prosocial behavior, future time perspective, self-transcendence meaning of life, China, adolescence

## Abstract

Awe is an emotion experience when individuals encounter with such powerful stimulate beyond their own understanding. Guided by conceptual analysis of awe as a trait positive emotion, we tested the hypothesis that dispositional awe results in an improvement of individual's self-transcendence meaning of life (STML) and affects future time perspective, and increase prosocial behavior. Mediational data demonstrate that the effects of awe on prosociality are explained, by improving STML self and future time perspective. These findings indicate that awe may help situate individuals within broader social contexts and enhance collective concern.

## Introduction

People usually experience a sense of awe when they encounter scenes such as temples and waterfalls. The experience of a sense of awe accompanied by a decline in self-importance makes people pay more attention to their role in society (Piff et al., 2015). Awe is an emotional experience felt when individuals encounter powerful stimuli that are beyond their own understanding (Keltner and Haidt, 2003; Tilquin, 2008; Piff et al., 2015); for example, a Taoist temple typically induces awe. In ancient China, Taoist temples^1^ were not only places for worshiping Taoist priests; they also served as almshouses. When a famine or natural disaster strikes, Taoist priests^2^ will always provide free accommodations and assistance for those who are homeless. Thus, it is important to examine why Taoist priests engage in so many prosocial behaviors that involve self-sacrifice and thus lead to greatness. These behaviors may be related to Taoist priests' long-term residency in Taoist temples and may be influenced by Taoist culture, which encourages Taoist priests to cultivate a sense of awe.

Awe is divided into state awe and dispositional awe, state awe can be induced in a certain way, such as watching magnificent natural landscape or extraordinary human masterpiece can both make individual experience awe (Van Cappellen and Saroglou, 2012); Dispositional awe is a personality trait that is stable across time and situation. There is a certain individual difference in experiencing awe, that is, individuals with dispositional awe are more likely to experience awe (Keltner and Haidt, 2003; Shiota et al., 2007). Researches have confirmed that, awe, as a positive emotional experience, is a contributing factor for prosocial behavior (Piff et al., 2015; Wang and Saudino, 2015; Prade and Saroglou, 2016; Ying et al., 2016; Bai et al., 2017). For example, when experiencing awe, individuals usually engage in more donating behaviors (Piff et al., 2015) and helping behaviors (Prade and Saroglou, 2016) and fewer attack behaviors (Ying et al., 2016) and antisocial behaviors (Bai et al., 2017). Dispositional awe strongly increases a person's sense of belonging to a larger group, which results in altruistic motivation (Shiota et al., 2007). Additionally, some studies have proven that dispositional awe predicts a person's generosity in economic decision-making games (Piff et al., 2015). People with higher levels of dispositional awe tend to contribute more money in economic games. Therefore, we speculate that dispositional awe may play a vital role in affecting prosocial behaviors; however, the underlying mechanism has yet to be elucidated.

The effect of dispositional awe on prosocial behaviors may be related to future time perspective and self-transcendence meaning of life (STML). Also, perception of future time may be another important factor in the effect of awe on prosocial behaviors (Chen, 2015). After experiencing awe, people are more likely to scruple future consequences of their behavior, and pay more attentions to others' needs without harming others' interests (Rudd et al., 2012; Piff et al., 2015; Bai et al., 2017). Thus, the present study aims to examine the direct effect of dispositional awe on prosocial behaviors and to determine whether STML and future time perspective mediate this effect.

In addition, STML refers to the transcendence of self (not self-centered), and presented as a transcendental attitude toward life (Li, 2006). STML is based on Buddhism's “remove thought of focus on self” and Taoism's “attitude toward loss and gain” (Li, 2006). The former refers to removing attachment to the self and reducing concern for the self, because the Buddhist view holds that obsessing self is the root of all suffering, and the latter means that everything in the world is dialectical and regular, so success or failure and gains and loss are also dialectical and meaningful. Because self- transcendence meaning of life to emphasizes removing attention from the self, which is beneficial for individual to turn their attentions to others, and pay more attentions to others' needs, and show more prosocial behavior. Meanwhile, STML is deeply influenced by awe, because awe promotes STML (Ying et al., 2016; Vötter and Schnell, 2019), therefore, STML may be another potential path for awe influencing prosocial behavior.

## Literature Review and Theory Foundation

### Dispositional Awe and Prosocial Behaviors

Prosocial behaviors are actions that individuals engage in to benefit others or the collective; such actions include self-sacrifice, donating, helping behaviors, and sharing (Penner et al., 2005; Bartlett and Desteno, 2006; Eisenberg et al., 2015). As gregarious and social creatures, humans must cooperate with one another to better adapt to the environment and deal with unpredictable changes. Thus, determining the factors that promote prosocial behaviors is of great significance for human development and has become a hot topic in recent years (Hysek et al., 2014).

Awe is a positive emotion (Bonner, 2015) that is able to inspire “small self” by inducing a decline in self-interest and self-importance and shifting the individual's attention to others and the collective (Piff et al., 2015; Bai et al., 2017). Additionally, awe induces “accommodation,” i.e., it makes people feel that they belong to a larger group and motivates them to engage in behaviors that benefit others (Keltner and Haidt, 2003; Piff et al., 2015; Bai et al., 2017), thus promoting prosocial behaviors.

First, awe induces changes in self-concept that make individuals change their cognitive behavior tendencies (Piff et al., 2015; Prade and Saroglou, 2016; Ying et al., 2016; Bai et al., 2017). Awe inspires “small self,” in which the self-concept becomes smaller and individuals view themselves less important and as part of a larger collective. Notably, small self is different from negative self; small self refers to a decline in self-concept, but negative self is a denial of the self (Piff et al., 2015). Individuals with high levels of dispositional awe are inherently less self-important, which makes them turn their attention to the greater collective (Shiota et al., 2007), decrease self-centered tendencies, increase collective identity (Piff et al., 2015; Bai et al., 2017), and engage in more prosocial behaviors.

Another feature of awe is accommodation. The word “accommodation” comes from Piaget's terminology; it refers to a process where in individuals reconstruct their cognitive structures when a new experience cannot be adapted to their original mental schema (Piff et al., 2015). When faced with new things, people with high levels of dispositional awe can change their mental schemas more easily and adapt to new things or environments (Shiota et al., 2007). In addition, some studies have noted that people experience awe when confronted with powerful experiences (such as works of art, giving birth, and nature) (Abdulwahid et al., 2014; Elk et al., 2016; Guan et al., 2018) or when they think they are in subordinate positions (Keltner and Haidt, 2003; Bai et al., 2017); under such circumstances, they pay less attention to their own benefits, and help others instead. Therefore, the present study speculates that dispositional awe is beneficial for promoting prosocial behaviors, and we put forward hypothesis 1:

Hypothesis 1: Individuals with high levels of dispositional awe show more prosocial tendencies.

### Mediating Effect of Future Time Perspective in Behaviors.

Time perspective is defined as a nonconscious process whereby the continual flows of personal and social experiences are assigned to time frames to give those events order, coherence, and meaning. Future time perspective is a kind of future-oriented behavior model (Zimbardo and Boyd, 1999; Stolarski et al., 2015; Rudolph et al., 2018). High future time perspective individuals can resist the temptation of short-term interests and obey longer-term goals (Carstensen et al., 1999; Chang et al., 2015; English and Carstensen, 2016; Wang et al., 2017; Rudolph et al., 2018), future time perspective was proved an important predictor for prosocial behavior (Moore et al., 2010). According to the theory of social dilemma, engaging in prosocial behavior will satisfy the needs of the group or others at the expense of self-short-term benefits; but in the long run, individuals who engage in prosocial behavior may also benefit from it (Van Lange et al., 2013). Therefore, consideration for future outcomes (such as future time perspective) is a guarantee that prosocial behavior can be implemented (Graaff et al., 2018; Nostrand and Ojanen, 2018).

Future time perspective may be a potential path for dispositional awe influencing prosocial behavior. Awe will affect the individual's time perception, individuals strongly experience awe will feel time more available (Rudd et al., 2012). According to socioemotional selectivity theory (SST), when an individual's sense of time is extended, they will be more motivated to engage in long-term outcomes, such as learning new knowledge, helping others, etc. Compared to low dispositional individuals, high dispositional awe individuals are more likely to experience a positive, obedient awesome experience that reminds them to focused on future outcomes and to act without harming others' interests (Wang et al., 2017). Based on this speculation, future time perspective may mediates dispositional awe influencing prosocial behavior.

Hypothesis 2: Dispositional awe can indirectly influence prosocial tendencies through future time perspective.

### Self-Transcendence Meaning of Life as a Mediator

Self-transcendence meaning of life (STML) refers to transcendence of self (not self-centered), and individuals believe that gain and loss are dialectical, inevitable and meaningful, presented as a transcendental attitude toward life that is focus others more than themselves and not afraid of loss (Li, 2002, 2006). The two core traits of self-transcending meaning of life (transcendence of self, not afraid of loss) are significantly related to prosocial behavior. First, high STML individuals present a not self-centered cognitive trait, that is, they reduce preference or concern for self-interest in interpersonal interaction. Studies found that reducing self-importance or maintaining attention to others' needs is beneficial for promoting prosocial behavior (Dambrun and Ricard, 2011; Dou et al., 2018; Maksim and Christin-Melanie, 2018). Second, high STML individuals also show that they are not afraid of losing, and believe that loss is meaningful. This transcendental concept of gains and losses can encourage individuals to understand and accept their own sacrifices engaging in prosocial behavior while helping others (Graaff et al., 2018). In summary, those high STML individuals are often more willing to contribute and show higher life values and life goal and choose to abandon self-interest to satisfy collective interests, which is an important embodiment of high-life meanings' value externalization (Brassai et al., 2011; Vötter and Schnell, 2019).

Studies have confirmed that a sense of awe will be accompanied with a strong self-transcendence emotional experience, enabling individuals to pursue spiritual world and become more altruistic (Prade and Saroglou, 2016; Ying et al., 2016; Chirico et al., 2017). For example, individuals are more willing to consume spiritual products after inducing awe (Haugan et al., 2012, 2014; Van Cappellen and Saroglou, 2012; Chirico et al., 2017), and pursuing spiritual would drive individuals to be more altruistic (Keltner and Haidt, 2003; Chirico et al., 2017). High dispositional awe people own a strong self-transcendence experience because they are more likely to experience awe. Out of this positive self-transcendence awareness and belief that encourages individual not be self-centered and pay more attentions to others' current difficulties and needs, and generate altruistic motivation (Lin, 2018; Vötter and Schnell, 2019), and promote individuals to engage in social activities that transcend the meaning of life so as to achieve spiritual satisfaction through helping others or engaging in charity (Binder and Freytag, 2013; Vötter and Schnell, 2019). According to this speculation, STML mediates dispositional awe and prosociality.

Hypothesis 3: Dispositional awe may also indirectly influence individuals' prosocial tendencies through self-transcendence meaning of life.

### The Current Study

**Figure 1:**
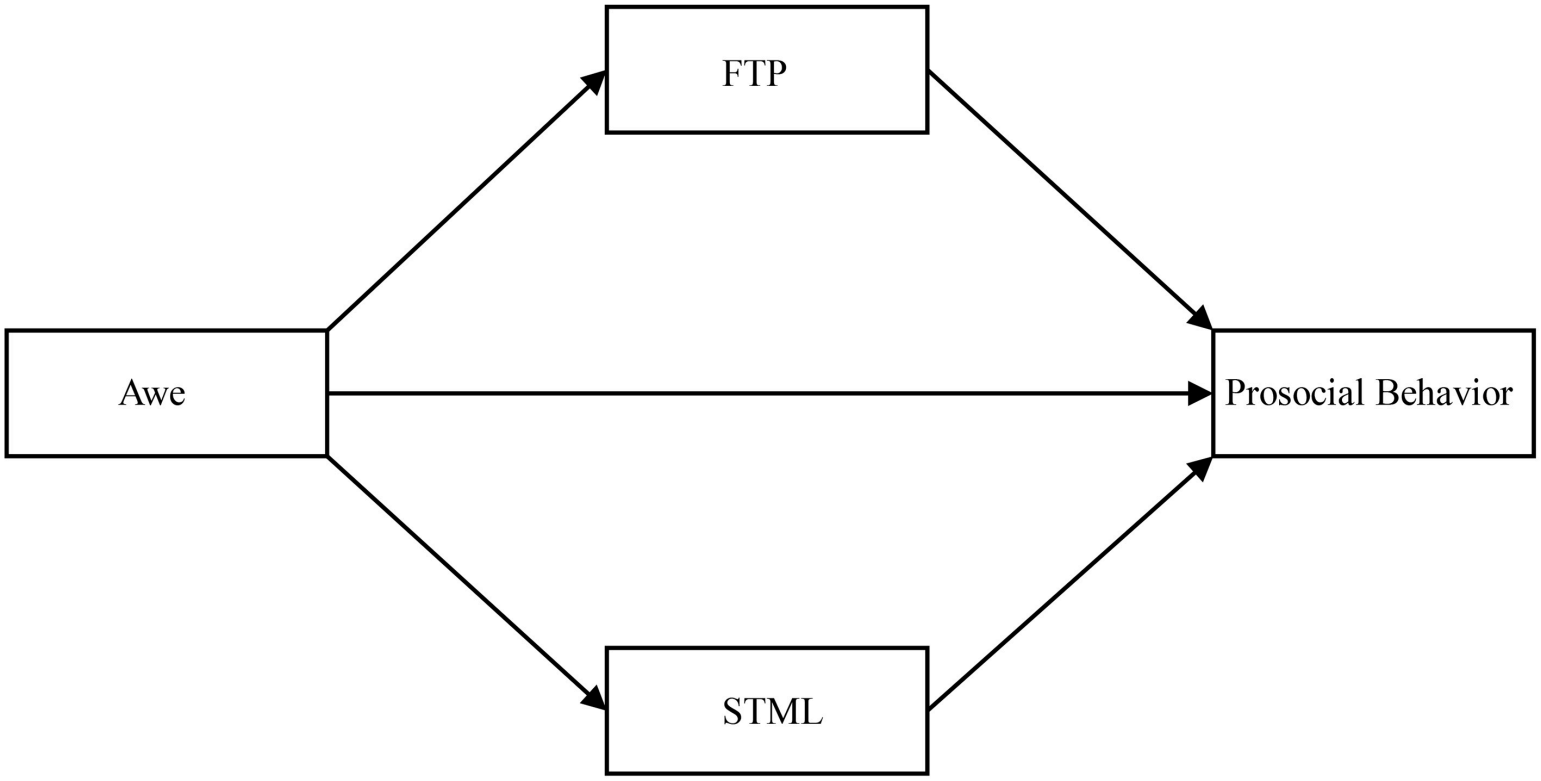
Theoretical model. FTP, future time perspective; SML, Self-transcendence Meaning of Life.

Dispositional awe emphasizes the individual differences in people's experiences of awe in the external world. Individuals who are high in dispositional awe are more likely to become less self-centered, thereby reducing the pursuit of personal interests and encouraging prosocial behavior. This dynamic may be related to STML and future time perspective. On the one hand, awe will influence individual's attitude toward future, and make they engage in behaviors that are more beneficial for long-term interests, such as establishing long-term relationships through prosocial behavior. On the other hand, awe may also motivate people to pursue a spiritual world that transcends the meaning of life, thereby reducing self-importance, strengthening their connection with the surrounding environment, and benefiting others. To examine the above hypotheses, this study used college students to test a dual mediation hypothesis model (Figure 1) in which future time perspective and STML mediate the effect of awe of prosocial behavior.

## Methods

### Participants and Procedure

A total of 3,347 college students (1,660 males and 1,687 females) from 2 universities in Guangdong Province of China participated in this research. There were 956 students from science and engineering colleges (such as the School of Computer Science) and 2,391 students from human and social science colleges (such as the School of Business Administration). There were 753 freshmen, 900 sophomores, 843 juniors, and 851 seniors.

First, the researchers distributed questionnaire notices and test schedules to 18 colleges (12 liberal arts colleges, 6 science academies) participating in the survey. Second, according to the research plan, the test was conducted collectively in class, all of the students were required to fill in written informed consent and return them. Finally, the questionnaire was carefully checked to eliminate invalid responses. All the participants received a small gift for their participation. A counselor or class teacher was trained to proctor the test. The training content included the test instructions and precautions. All the procedures were in accordance with the ethical guidelines of the American Psychological Association (APA) and were approved by the Institutional Review Board (IRB) at School of Education, Guangzhou University.

### Measures

#### Awe

The awe subscale of the *Dispositional Positive Emotion Scales (DPES)* (Shiota et al., 2006) was used to assess the participants' dispositional awe. The subscale included 6 items. The participants rated their agreement with each statement on a 7-point Likert scale ranging from 1 (completely disagreement) to 7 (complete agreement). Representative items like “I often feel awe” and “I see beauty all around me.” In the present research, the Cronbach's α for dispositional awe was 0.78. Higher scores indicate that individuals are more likely to experience awe.

#### Prosocial Behavior

The Prosocial Tendencies Measure (PTM) was developed by Carlo and Randall (2002), revised by Kou et al. (2007), and used to assess university students' prosocial behavior in the present study. The PTM consists of six dimensions: **public** (4 items, Cronbach's α = 0.75; e.g., “I can help others best when people are watching me”), **anonymous** (5 items, Cronbach's α = 0.79; e.g., “I prefer to donate money anonymously”), **dire** (3 items, Cronbach's α = 0.77; e.g., “I get the most out of helping others when it is done in front of others”), **emotional** (5 items, Cronbach's α = 0.77; e.g., “It is most fulfilling to me when I can comfort someone who is very distressed), **compliant** (5 items, Cronbach's α = 0.78; e.g., “When people ask me to help them, I don't hesitate”), and **altruism** (4 items, Cronbach's α = 0.65; e.g., “Most of the time, I help others when they do not know who helped them”). The participants were asked to rate the extent to which the statements described themselves on a 5-point scale ranging from 1 (*Does not describe me at all*) to 5 (*Describes me well*). The average score for the 26 items was calculated, with a higher score indicating a higher level of prosocial behavior. The Cronbach's alpha for this scale was 0.92.

#### Future Time Perspective

Future time perspective was measured by the 13-item Future Time Perspective subscale in the Zimbardo Time Perspective Inventory (ZTPI_F), which was developed by (Zimbardo et al., 1997; Zimbardo and Boyd, 1999)). This scale was translated using a back-translation procedure. This study has extracted a total of 13 questions for future factors as a future time perspective questionnaire. The score for the Future subscale suggests that behavior is dominated by striving for future goals and rewards. Examples of Future questions include “I am able to resist temptations when I know there is work to be done” and (negatively) “I take each day as it is rather than trying to plan it out.” The participants rated their agreement with each statement on a 5-point Likert scale ranging from 1 (*strongly disagree*) to 5 (*strongly agree*). The Cronbach's α for this scale was 0.78.

#### Self-Transcendence Meaning of Life

Self-transcendence meaning of life was measured by the Self-transcendence Meaning of Life Scale (SMLS), which was developed by Li (2002, 2006). This scale consists of 8 items, 8 items are based on two aspects: first, the concept definition and operational definition of self-transcending meaning of life; second, the results of in-depth interview research. The SMLS consists of two dimensions: obtain the meaning of the failure (GMF, 3 items, Cronbach's α = 0.73; items like “I can sense that life is rich in losses.”) and detachment from success or failure (DSF, 5 items, Cronbach's α = 0.74; items like “Loss may be more meaningful than gain in life”). This scale rated on a 4-point scale, from 1 (*strongly disagree*) to 4 (*strongly agree*), where higher scores reflect greater STML, no reversed scoring. The Cronbach's alpha for this scale was 0.81.

### Data Analysis

SPSS21.0 and Mplus 7.0 were used to analyze the data. First, we used correlation analyses to explore the correlations between the main variables. Second, we tested hypotheses using structural equation modeling (SEM), and demographic variables (gender and grade) were considered covariates in the models. Finally, we used mediation analyses to examine theoretical model, and then used a robust method of maximum likelihood because the dependent variable was ordinal. An RMSEA value below 0.08 and a CFI value over 0.90 indicate a good fit (Maccallum et al., 1996). In addition, bootstrapping we used is 5,000 samples, and a 95% confidence interval (CI) was used to determine whether the mediation effect was significant. When the 95% CI for an indirect effect did not include 0, the indirect effect was significant.

## Results

### Descriptive Statistics and Correlation Analyses

Table 1 shows the means, standard deviations, and correlation coefficients. Among the variables, awe was positively associated with future time perspective (*r* = 0.41, *p* < 0.001), STML (*r* = 0.35, *p* < 0.001), and prosocial behavior (*r* = 0.42, *p* < 0.001). Future time perspective (*r* = 0.45, *p* < 0.001) and STML (*r* = 0.44, *p* < 0.001) were also positively associated with prosocial behavior.

**Table 1:** The means, standard deviations, correlations, and reliabilities among the variables.

***Variable***	***M***	**SD**	**1**	**2**	**3**	**4**	**5**
1 Gender	0.50	0.50					
2 Grade	1.46	1.10	−0.13^***^				
3 Awe	4.61	0.95	−0.03	0.06^**^			
4 Future time perspective	3.61	0.50	0.11^***^	−0.06^**^	0.41^***^		
5 Self-transcendence meaning of life	3.04	0.51	0.06^**^	−0.07^***^	0.35^***^	0.38^***^	
6 Prosocial behavior	3.60	0.52	0.06^**^	−0.05^**^	0.42^***^	0.45^***^	0.44^***^

*N = 3,379*.

** p < 0.01;

*** *p < 0.001*.

We used Mplus 7.0 to create a latent variable for prosocial behavior by using the six dimensions: public, anonymous, dire, emotional, compliant, and altruism. We then used SEM to examine the relationship between awe and prosocial behaviors. The results showed that awe was positively associated with prosocial behavior (β = 0.44, *p* < 0.001). This finding supported our first hypothesis.

### Testing the Indirect Effect of Future Time Perspective and Self-Transcendence Meaning of Life

We used SEM (bootstrapping with 5,000 samples) to determine whether future time perspective and STML (latent variables are divided into two dimensions: obtain the meaning of the failure and detachment from success or failure) simultaneously mediated the effect of dispositional awe influencing prosocial tendencies. The fit indices were $\chi_{\left(63,\text{N}=3379\right)}^{2}$ = 652.92, RMSEA = 0.08 (90% CI = [0.08, 0.09]), CFI = 0.91, and SRMR = 0.04, indicating that the model was a good fit. Consistent with our hypothesis, future time perspective was a significant mediator (indirect effect = 0.10, SE = 0.01, *p* < 0.001), and the 95% CI for the indirect effect (0.08, 0.12) did not include zero. Awe was positively associated with future time perspective (β = 0.41, *p* < 0.001), and future time perspective was positively associated with prosocial behavior (β = 0.25, *p* < 0.001)^3^.

**Figure 2:**
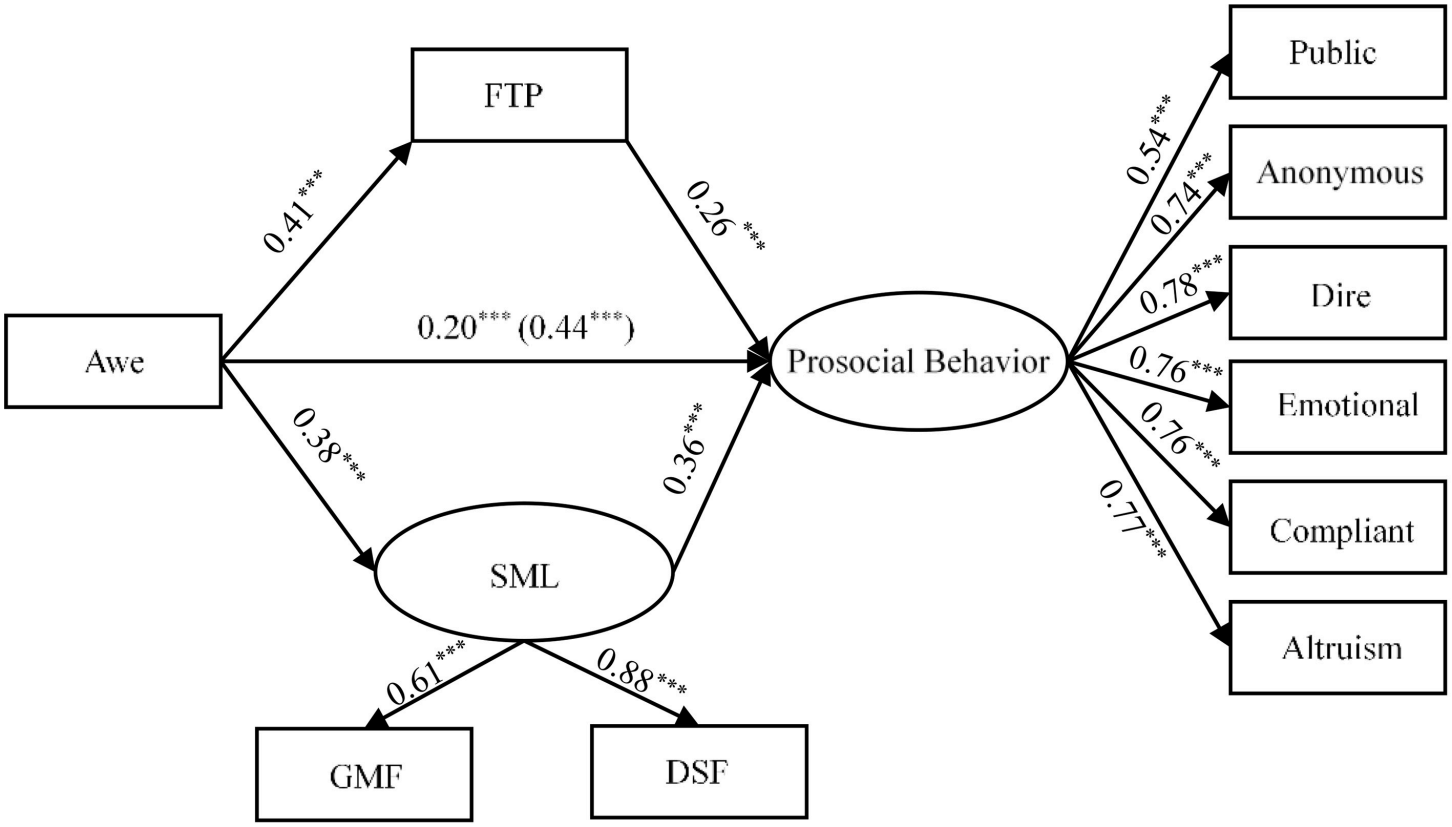
The indirect effect of FTP and SML in the relationship between Awe and Prosocial Behavior. Standardized coefficients are reported; ***p < 0.001; FTP, future time perspective; SML, Self-transcendence Meaning of Life; GMF, Got the Meaning of the Failure; DSF, Detached from Success or Failure.

As expected, STML significantly mediated the positive association between awe and prosocial behavior (indirect effect = 0.14, SE = 0.01, *p* < 0.001), and the 95% CI for the indirect effect (0.11, 0.16) did not include zero. Awe was positively associated with STML (β = 0.38, *p* < 0.001), and STML was positively associated with prosocial behavior (β = 0.35, *p* < 0.001). After accounting for the mediating variables of future time perspective and STML, the direct effect of awe on prosocial behavior decreased from β = 0.44 (*p* < 0.001) to β = 0.21 (*p* < 0.001). The model results are shown in Figure 2.

In addition, we performed multiple mediator analysis to determine which mediators had a larger effect; we found no significant difference between future time perspective and STML.

## Discussion

Awe is a positive emotion that has been suggested to promote prosocial behavior and foster emotions such as happiness and love (Light et al., 2015). The present study verified this speculation in a group of Chinese late adolescents, and we found that adolescents with high levels of dispositional awe exhibited more prosocial tendencies. In addition, the present study found that dispositional awe can not only directly influence prosocial behavior but can indirectly influence prosocial behavior by promoting an individual's future time perspective and enhancing STML. This result elucidated the possible mechanism of awe's influence on prosocial behavior, from the perspective of life meaning and time frame, thereby advancing related research on the relationship between positive emotions and prosocial behavior.

### Theoretical Implications

The theoretical contributions of this research are threefold. First, from the perspective of dispositional awe, the present study claimed that awe has a positive influence on prosocial behavior in Chinese late adolescents and confirmed the prior assumption that “awe promotes prosocial behavior” (Piff et al., 2015; Ying et al., 2016). This result is not just a supplement to the study of the relationship between induction awe and prosocial behavior but further elucidates the possible existed mechanism for maintaining and promoting prosocial behavior. Compared to prior studies on the relationship between state awe and prosocial behavior (Prade and Saroglou, 2016), the effect of dispositional awe on prosocial behavior is more consistent across time and context; thus, cultivating young people's awe is also an important way to standardize behaviors in line with social ethics. As a recent finding stated, social mindfulness—a focus on the current interests of others shown by behaviors during interpersonal interactions—can effectively inspire cooperative behaviors (Dou et al., 2018). Thus, individuals with high levels of dispositional awe are more likely to perceive awe, they will think more about others when making decisions, they will engage in prosocial behavior that does not harm others, and they will protect others' interests.

Second, future time perspective mediates the effect of dispositional awe on prosocial behavior. That is, individuals with high levels of dispositional awe are more inclined to weigh the potential consequences of their future behaviors, make decisions that maximize long-term benefits, and sacrifice their short-term self-interest to satisfy others. This finding provides evidence that future orientation theory may explain the effect of dispositional awe on prosocial behavior. According to the perspective of future orientation theory, individuals with high levels of consideration for the future tend to use long-term goals to guide their behaviors, and they are not easily influenced by short-term gains. In contrast, individuals with low levels of future consideration focus more on current than long-term benefits (Stolarski et al., 2015). Thus, if an individual focuses on future outcomes, he or she will engage in activities that will be beneficial in the future rather than accounting for the gains and losses of immediate benefits (Wang et al., 2017). Future time perspective is a stable and reliable personality trait that reflects individuals' preferences for determining their own behavior using a short-term or long-term time frame (Kozik et al., 2015). Individuals with high levels of dispositional awe due to consideration of behavior consequences, high dispositional awe individuals are more likely to weigh current behavior from long-term interests, which is beneficial for individuals regulating their own behavior without harming others' interests (Stolarski et al., 2015).

More importantly, another contribution of this research is that we further confirm the dual mediating effect between STML and future time perspective in dispositional awe and prosocial behavior is also significant, indicating that prosocial behavior can be simultaneously influenced by both future orientation and attitude toward life (self beyond the meaning of life).That is, STML not only helps to reduce poor adaptation but also promotes mental health and prosocial behavior (Triplett et al., 2012; Woo and Brown, 2013; Steger et al., 2015; Wang et al., 2016). STML is based on the concept of a Chinese native religion (Taoism) and Buddhist culture, and it includes two dimensions: the acquisition of meaning from failure and the adoption of the transcendental meaning of success or failure. Therefore, individuals with a strong sense of STML are more likely believe that success and failure are inevitable and meaningful and to show a transcendental attitude toward life. This result expands the mechanism of diminished self-centeredness in explaining the relationship between awe and prosocial behavior (Piff et al., 2015; Bai et al., 2017). Keeping a transcendental attitude that transcends life's meaning is closely related to the concept of “diminished self” (Ganellen and Blaney, 1984). An individual with a diminished self has reduced self-importance and turns their attention to the larger collective; thus, individuals with a high STML tend to pay less attention to themselves and instead turn their attention to others in an altruistic manner.

### Practical Implications

The findings of this study have certain educational implications for improving adolescents' prosociality.

First of all, keeping awe is a self-constrained behavior individuals. If human beings are not afraid of anything, they will be self-centered and even commit illegal activities. Teenagers are at a critical stage of personality development, at this period, they grow rapidly but mental develop slowly, and is not only a period of unstable psychological age, but also a frequent period of problem behavior. Therefore, it is undoubtedly very important for young people to cultivate awe.

Second, young people are encouraged to weigh their behavior according to a future-oriented time frame. In the long term, a future time perspective can enhance and sustain cooperative behavior and contribute to individuals' future plans. This is of great practical significance not only for personal life decision making and class management but also for regulating and guiding positive collective behavior. Because future time perspective is so important, it should be incorporated into educational programs. In addition, effective training methods that comprehensively use behavioral training, psychological counseling, and other strategies should be developed to help young people establish and maintain an “other-focused” social cognition style.

Third, STML can be included in education programs regarding adolescent mental health or lifestyle. STML emphasizes that success and failure are inevitable, meaningful and mutually transformative and facilitates a transcendental attitude toward life. For contemporary teenagers, stress is a common experience. As they get older, the exams approach, and pressures come one after another. Stress is a psychological problem that plagues young people. In the process of guiding young adolescents to learn psychological adjustment, STML can be treated as a method for psychological adjustment; because it emphasizes a “transcendental” life attitude, it is helpful for alleviating adolescents' psychological problems.

### Limitations and Future Research

The current study reveals that dispositional awe promoted prosocial behavior in late Chinese youth through the mediating mechanism of future time perspective and STML and provides a theoretical basis for exploring prosocial behavior's promotion and maintenance mechanism. However, it should be noted that this study has shortcomings.

First, the present research lacks sufficient persuasiveness in explaining the causal relationship between dispositional awe and prosocial behavior because of the use of cross-sectional data. In addition, the assessment tools used in this study mainly rely on self-reporting. Although this study reduces the interference of common method bias by emphasizing anonymity, it is also difficult to avoid the impact of social approval. Therefore, future research could employ a longitudinal design that includes multiple data-collection methods (such as self-assessment combined with others' evaluations) and time points, thus providing a deep examination of the causal relationship between dispositional awe and prosocial behavior.

Second, from the perspective of the research objective, this study mainly used a convenient sampling method, and research was carried out on late-adolescent college students. Therefore, the conclusions of this study cannot be generalized to all youths or adults. Future research can further increase the ecological validity of these findings by expanding the age range of the sample and the group type of the sample (e.g., students, employees, volunteers).

## Conclusions

1. Individuals with high levels of dispositional awe show more prosocial tendencies.
2. Dispositional awe can indirectly influence prosocial tendencies through future time perspective.
3. Dispositional awe may also indirectly influence individuals' prosocial tendencies through STML.
4. This study has certain enlightenment to education. It can encourage young people to balance their behaviors from the future time frame by cultivating awe, and put STML or life education into adolescent mental health education content system so as to promote youth development.

## Ethics Statement

This project is approved by the Ethics Review Committee (IRB) of Education School, Guangzhou University, accords with the principle of ethics. All participants have filled written informed consent before study.

## Author Contributions

J-JL, KD, and Y-GN: conceptualization. Y-JW and KD: data curation. J-JL and KD: formal analysis, methodology, software and writing—original draft. KD and Y-GN: investigation. J-JL and Y-JW: project administration. KD, Y-JW, and Y-GN: resources. Y-GN: supervision. Y-JW and Y-GN: writing—review and editing.

### Conflict of Interest Statement

The authors declare that the research was conducted in the absence of any commercial or financial relationships that could be construed as a potential conflict of interest.

## Footnotes

^1^Taoism is a native religion of China and is closely related to Chinese culture. It is a religion that values life and emphasizes the concept of equal life, emphasizes that “tangible is valuable,” and considers “Dao” the highest form of faith. Taoist temples are places for Taoist practice.

^2^A Taoist is a cleric of Chinese Taoism. Taoists preach and do their best for their religion while fulfilling their own practice.

^3^We tested an alternative model of sequential mediation model (future time perspective as first mediator and self-transcendence meaning of life as second mediator), and the results showed poor and unacceptable model fit: χ2_(47, N = 3379)_ = 1253.92, RMSEA = 0.09 (90% CI = [0.08, 0.09]), CFI = 0.86, and SRMR = 0.09.
